# Art Meets Sport: What Can Actor Training Bring to Physical Literacy Programs?

**DOI:** 10.3390/ijerph17124497

**Published:** 2020-06-23

**Authors:** Lisa M. Barnett, Rea Dennis, Kate Hunter, John Cairney, Richard J. Keegan, Inimfon A. Essiet, Dean A. Dudley

**Affiliations:** 1 Institute for Physical Activity and Nutrition, School of Health and Social Development, Deakin University, Geelong 3220, Australia; 2 School of Communication and Creative Arts, Deakin University, Geelong 3220, Australia; rea.dennis@deakin.edu.au (R.D.); k.hunter@deakin.edu.au (K.H.); 3 School of Human Movement and Nutrition Sciences, University of Queensland, St Lucia 4067, Australia; j.cairney@uq.edu.au; 4 Research Institute for Sport and Exercise (RISE), Faculty of Health, University of Canberra, University Drive, Bruce 2617, Australia; Richard.Keegan@Canberra.edu.au; 5 School of Health and Social Development, Deakin University, Geelong 3220, Australia; iessiet@deakin.edu.au; 6 Centre for Sports, Exercise and Life Sciences (CSELS), Faculty of Health and Life Sciences, Coventry University, Coventry CV1 5FB, UK; 7 Macquarie School of Education, Macquarie University, Sydney 2109, Australia; dean.dudley@mq.edu.au

**Keywords:** child, physical education, actor training, theatre, performance, performing art

## Abstract

The aim of this communication is to highlight synergies and opportunities between the fields of education, sport and health and the performing arts for the promotion of physical literacy. First, physical literacy is introduced and then defined according to the definition used in this communication. Secondly, we highlight the gap in physical literacy interventions, in that they do not address learning based on a holistic comprehensive definition of physical literacy. Then we provide examples of interventions that do borrow from the arts, such as circus arts, and show how these approaches explicitly link to the discipline of arts. This is followed by program examples, which approach motor and language development from discipline-specific perspectives. Then we introduce actor training (within the discipline of arts) in terms of how this approach may be useful to our understanding of physical literacy and how to expand the conception of physical literacy to include affective meaning making, and tolerance for ambiguity and discomfort in not-knowing. Finally, we conclude with the next step for the bridging of disciplines in order to further our journey to understand and improve physical literacy.

## 1. Introduction

The concept of physical literacy has generated substantial discussion and new research in the fields of health, sport and education [1]. The notion of embodiment is central to early definitions of physical literacy, where this state can be described as one’s mental and physical body being in harmony together, as one, and also with one’s surroundings [2]. The more recent Australian definition and framework of physical literacy included four domains (physical, psychological, social and cognitive) and 30 ‘elements’ across these four domains to characterise the diverse capabilities that combine to comprise physical literacy. The framework also encourages a strong focus on physical literacy as a lifelong learning journey [3,4]. This paper will highlight that few programs aiming to improve physical literacy have drawn from all four domains in the framework. Other fields, such as performance and theatre studies, may be able to offer insights into understanding physical literacy, and how to improve it, for example, through actor training. Actor training is a comprehensive methodology which—when undertaken as a rigorous, consistent and regular practice—evokes an expanded cognitive experience encompassing the senses, perception, imagination and physicality [5]. In this paper, the Australian physical literacy framework is used to illustrate how the practices of actor training can be used to both enhance our understanding of physical literacy, and offer new avenues for promoting and developing physical literacy across the lifespan. The end goal of the paper is that those in education, sport and health, realise the synergies and opportunities between those fields and the performing arts—particularly when it comes to developing diverse and enriching movement experiences: endorsed for the promotion of physical literacy.

## 2. The Embodied Person as Core to Physical Literacy

Whitehead reflected on the concept of embodiment and physical education, suggesting that the discipline of physical education tended to view the body from a dualistic position: i.e., that the body is situated as separate to other aspects of the person [2]. In contrast, the ‘holistic’ person is embodied, in that the body seen as integrated with other aspects of the person, and particularly with the world around them. Whitehead argued that we exist through our relationship with the world, rather than in isolation. She suggested that through the body and movement, we understand and react to the world around us, fulfilling our inherent needs to pursue growth, control, connection and thriving. Whitehead’s influential proposal was that the goal of physical education has been achieved when one is in harmony with their surroundings, and integration of their mental and physical body is realised.

*“It is perhaps hard for those concerned with improving aspects of our physical attributes to accept that their ultimate goal is to enable pupils to disregard the complexities of bodily control and coordination in the pursuance of a close and articulate liaison with the world. The real value of the capacities of our embodied dimension is not realised in isolation from our surroundings but in intimate relationship with them”*.

[2]

This concept of embodiment is core to the concept of physical literacy. Whitehead [6] published a subsequent article in which she summarised philosophical views that support the notion of embodiment being pivotal to human life. She reasoned that the capacity to fully realise our embodied selves can be viewed as physical literacy, and she defined physical literacy as the “motivation, confidence, physical competence, understanding and knowledge to maintain physical activity at an individually appropriate level, throughout life” [6]. While popular and clearly grounded in an expansive analysis of philosophy and education, this definition has been criticised as too reductive/simplistic, and potentially simply a combination of four other previously existing constructs e.g., [7,8]. Alternatively, if we view this definition as an attempt at unification or synthesis of these four constructs (among others), which Cairney et al. [9] describe as an experiential convergence of these constructs as part of the ‘process’ of physical literacy, two interesting possibilities emerge. First, by linking these constructs together, there is at the least the potential for novel theoretical synthesis, such as the linking of theories in motivational psychology (e.g., self-determination theory) to theories of motor development (e.g., dynamic systems theory). Indeed, if we consider each construct having its own rich tradition of theory, then if these constructs are indeed conceptually linked, it will be important to explore possible underlying theoretical synthesis that in turn might help us to understand how the different constructs fit together. Second, this synthesis of constructs challenges us to think in terms of whether the whole is indeed greater than the sum of parts [10], or even if, perhaps more fundamentally, they do indeed ‘hang together’ in a single coherent model as the definition implies. If this is the case, then there is at least partial empirical support for the notion of a latent construct of physical literacy, comprised of these constructs [11].

As reflected in Whitehead’s definition, physical literacy arguably reflects convergences of theories in motivation, motor development and sport pedagogy. For example, regarding motivation, a recent narrative review [12] noted that most research in sport and motor development contexts adopted the intrinsic-extrinsic continuum of motivational regulation, offered by Deci and Ryan’s Self-Determination Theory (SDT) [13]. In this context, motivation is typically viewed as most optimal, and most sustainable, when learners perceive inherent value in the activity, and gain intrinsic rewards (such as enjoyment) from a task: with no need for external inducement or expectations. Likewise, most papers recommended that tasks and situations should seek to develop intrinsic motivation by supporting the core psychological needs specified in self-determination theory: the need for competence (to experience progression and success on a task); relatedness (the need to feel both friendship/affiliation and group membership/belonging); and autonomy (the need to experience choice and a sense-of-control). The emphases of physical literacy on the learner’s embodied experience and interaction with their environment can both directly correspond to SDT’s emphasis on subjective perceptions and creating environments that support (rather than thwart or frustrate) basic psychological needs. These experiences can occur away from sporting fields, tracks, courts and pools, and yet still engage development in the physical, cognitive, psychological and social domains—thus still representing physical literacy [14]. Similarly, physical literacy thinking is aligned to contemporary theories of motor development such as dynamic systems theory (DST). DST theory proposes that movement is produced from the interaction of multiple sub-systems within the learner, task and environment [15]. All of the sub-systems—processes, neural pathways and conditioned interactions with the environment—develop through experience and interaction to produce the most efficient movement solution for each specific task [15]. DST also proposes that no sub-system is most important in this process [16]. Rather, a small, but critical change in one sub-system can cause the whole system to shift, resulting in a new motor behaviour [17]. This phase shift, or transition period is critical to DST’s application to motor development. According to DST, development is a non-linear process [15]; suggesting that one’s physical literacy capability is not developed in a continuous manner—at a steady rate. Thus, teachers and practitioners need to consider and evaluate all aspects of the task, person, and environment when trying to develop someone’s physical literacy. The realisation that development is non-linear and heavily dependent on the richness of interactions between ‘sub-systems’ (within the learner as well as between learner and environment) also directly corresponds to physical literacy thinking emphasising individual journeys and non-normative assessment expectations [4]. To this end, we should not expect physical literacy to only be developed in sport and physical education, but in any activity that pushes us to extend our experiences and capabilities through engaging out physical embodiment. This realisation raises the possibility of actor training and performing arts being vital opportunities to promote physical literacy.

## 3. Physical Literacy in Australia

Since its re-proposal by Whitehead, the concept of physical literacy has inspired various initiatives and programs around the world, spanning sport, education and health [18]; but rarely in the arts. In Australia, the national sporting body (Sport Australia) led the push to develop a definition of physical literacy and framework for Australia [3] with involvement from researchers and practitioners from a wide range of disciplines and interest areas (i.e., Education, Sport, Public Health, Recreation, Disability, Psychology, and Indigenous [14]); previously identified as vested vital stakeholder groups [19]. The resulting Australian approach to defining physical literacy reflects the holistic, integrative nature and is becoming increasingly accepted in new initiatives across Europe and Oceania e.g., [20,21]. To adequately reflect the various—sometimes conflicting—principles and aims within physical literacy discourse, four defining statements were articulated, in consultation with an international panel of experts: (a) at its *core*: physical literacy is lifelong holistic learning acquired and applied in movement and physical activity contexts; (b) the *constitution* of physical literacy reflects ongoing changes integrating physical, psychological, cognitive, and social capabilities; (c) physical literacy is *important* because it is vital in helping us lead healthy and fulfilling lives through movement and physical activity; and (d) the *aspiration* or desirable configuration of physical literacy is when a person is able to draw on their integrated physical, psychological, cognitive, and social capacities to support health-promoting and fulfilling movement and physical activity—relative to their situation and context—throughout their lifespan. The approach explicitly identified four inter-related domains (physical, psychological, cognitive and social), and 30 elements within these domains. For example, ‘movement skills’ is an element in the physical domain, ‘motivation’ in the psychological domain, ‘rules’ in the cognitive domain and ‘collaboration’ in the social domain, (please see website for a full description of elements [22]. More specifically though, the framework identified elements other than sport and movement skills that have not previously featured in other definitions, that may be beneficially integrated into movement experiences to develop physical literacy. These included such elements as *connection to place*—reflecting an appreciation and connection to the environment, both built and natural, in relation to movement and physical activity; *self-regulation*, reflecting the ability to manage emotions and resulting behaviours in relation to movement and physical activity; *society and culture*—reflecting an appreciation of cultural values which exist within groups, organisations, and communities; and *collaboration*—reflecting social skills for successful interaction with others, including: communication, cooperation, leadership and conflict resolution [22]. Ongoing research to evaluate the implementation of the Australian physical literacy framework identified that sports coaches and administrators greatly appreciate the ‘reminder’ that their activities develop the whole person, and the ability to promote that and explicitly incorporate it into plans and activities [23]. Further, the identification of such elements as integral to physical literacy clearly opens up the possibility for various artistic activities, extending far beyond competitive and recreational sport and exercise. Nevertheless, with the exception of circus-based activities in Canada (see below), very few programs have explicitly linked the arts with physical literacy: until now.

## 4. Physical Literacy as Learning

Focussing on physical literacy as a construct of learning was first proposed by Dudley [24]. He proposed that observed behaviour change resulting from learning-based interventions and measured according to models used by learning theorists could capture ‘physical literacy’. Specifically, Dudley (2015) identified that neo-Piagetian understandings of learning and development (defining increasing complexity of each learning stage according to a child’s information processing system, instead of simply a logical progression) align well with the ‘Whiteheadian’ philosophical intent.

In the Australian definition of physical literacy, learning is a core consideration, as reflected by the ‘core’ defining statement. As such, for each of the 30 elements there is the notion of learning or progression across characteristic stages. In this way, anyone and everyone is on a physical literacy journey at any and every point in their lives. This journey is not always progressive and advancing—there may be times when we digress and regress due to injury, illness, life stages (such as pregnancy), or life circumstances (such as start or end of full-time work). The notion of learning is tied back to the Whiteheadian concept of embodiment, i.e., the core of our proposed definition of physical literacy is learning, which more fundamentally means any and all adaptations a person experiences in relation to being physically embodied [14]. The Australian approach also articulated how learning progresses, by drawing on an established learning framework identified by Dudley [24] (i.e., Structure of Observed Learning Outcomes (SOLO) Taxonomy [25]). In relation to physical literacy, and in an explicit attempt to use accessible and easily-understood terminology, the framework suggested five stages: *Pre-foundational*—wherein a person is experiencing, playing or exploring limited forms of movement; *Foundation and Exploration*—wherein a person is learning and exploring their capabilities for movement; *Acquisition and Accumulation*—wherein a person is frequently practicing and refining their capabilities for movement; *Consolidation and Mastery*—during which a person is able to perform and analyses their capabilities for movement; and finally *Transfer and Empowerment*—when a person is able to transfer their capabilities for movement to new and different situations [22]. The challenge then, is how to assist a person to move forwards in their physical literacy journey.

## 5. Programs in the Name of Physical Literacy

Empirical evidence supporting the effectiveness of physical literacy-based interventions/programs is lacking [26]. Of course, many interventions could fall under the remit of physical literacy if, at a minimum, one or more of the constructs comprising the definition are targeted. However, in this concept paper, we are more interested in interventions that have explicitly situated their programs as an effort to improve physical literacy. Some intervention studies, though referring to improving physical literacy, have focused specifically, or even exclusively, on fundamental movement skills e.g., [27,28,29]. For instance, in a protocol paper, a multilevel cluster randomised controlled trial aimed at improving young children’s (ages 3–5) healthy eating practices and physical literacy is described [28]. Here, data on pre-schoolers “physical literacy and gross motor skills” were collected using a standardised testing protocol for assessing fundamental motor skills. Similarly, the effectiveness of a nine week active classroom intervention in promoting the physical literacy of Year 5 pupils (aged 9–10) in the United Kingdom is evaluated based on children’s ability to perform fundamental movement skills [29]. Fundamental movement skills, though integral to physical literacy development, are just one aspect of this holistic concept—captured by only a few of the 30 elements identified within the Australian framework. That is not to dismiss fundamental movement skills, as there is good evidence that developing them leads to increased subsequent uptake of physical activity [30], as well as increased fundamental movement skills [31,32].

Other interventions move beyond fundamental movement skills but still stay within the physical domain. For example, an eight-week program on elementary-aged children’s physical literacy: reporting significant improvements in cardiovascular endurance, balance and stability skills, participation in diverse environments, and interest in participating in diverse activities [33]. When seeking to develop measures to assess physical literacy, it is important to not just focus on the physical domain, or only one or two elements of physical literacy (e.g., movement skills) [34]. Some physical literacy interventions have targeted domains other than the physical. In university students, an 11-week physical literacy intervention constituted weekly sessions of various movement-based activities which targeted a number of elements of physical literacy (movement competence, motivation, confidence, knowledge and understanding) [26]. Overall, physical literacy levels were reported to improve along with some elements (e.g., motivation, knowledge and understanding) of physical literacy. Similarly, another study in adults evaluated physical literacy in terms of: physical activity behaviour; attitude towards an active lifestyle; motivation towards activity; knowledge and self-confidence/self-efficacy [35]. In the aforementioned studies, a consistent theme is the focus on only a few elements of physical literacy, which is not consistent with Whitehead’s emphasis on a holistic definition/understanding. Physical literacy interventions designed with the intention of addressing *most or all* the 30 elements identified by the Australian physical literacy framework—or even all four learning domains within the Sport Australia Framework—are lacking.

## 6. Physical Literacy Interventions that Borrow from the Arts

Recognising that physical education still remains rooted in an exercise/sport model of delivery, which may be off-putting to some students and therefore negatively impact both participation and enjoyment [36], researchers and practitioners are increasingly interested in alternative forms of activity that may be more inclusive. Circus arts instruction is one such example. Circus arts instruction encourages movement skill development through artistic expression and group-based movement, incorporating all of the major disciplines of circus arts: acrobatics, manipulation, equilibrium, aerials, and clowning. The nature of skills (with and without equipment/implements) are very different to that of traditional sports. Participants are encouraged to acquire movement competences across these disciplines using equipment such as diablo, rola bola, pogo sticks, trapeze as well as flower sticks, pins, balls, ropes and silks.

In circus (as in other expressive arts), the participant learns to perform “for” an audience [37]. In circus, creativity and expression through movement to convey feelings or states are practiced and encouraged; similar to a dance performance. The artistic and performance-based elements of circus, in conjunction with the physical competencies required to master different disciplines, require creativity, expression, risk-taking, trust, perseverance, and discipline [38,39]. The skills are mastered for performance: i.e., to: tell a story, create a mood, or express an idea or emotion, or play a character. In other words, in circus arts, it is not enough that a child knows how to juggle; what is important is the ability to juggle to others as a form of expression. Therefore, there is a relationship between the performer and the audience, in terms of how the performance is received and interpreted by the audience. Mirroring sport and exercise movements, the novelty of the activities across disciplines may result in progressions that are variable across students (some pick up the skills and progress more rapidly compared to others). Virtually all students in such studies reported little or no exposure to skills, which means it can be viewed as an even playing field. Moreover, the diverse range of activities, from clowning to juggling to acrobatics, means there is ‘something for everyone’ and therefore more than one skill progression pathway. It is for all these reasons that circus arts instruction is viewed as particularly relevant to use for the development of physical literacy as it emphasises skill development, confidence and competence, as well as fun and social participation using activities that are challenging, engaging, novel and quite distinct from each other [40].

One study has evaluated the use of circus arts instruction as an alternative pedagogy to standard physical education classes in Montreal, Canada to achieve physical literacy [40]. Children in the circus arts instruction intervention (content through the National Circus School in Montreal) were compared to children receiving standard curricular physical education using the PLAYfun [41] and PLAYself tools. Children receiving circus arts instruction showed greater improvements in competence, confidence and comprehension of movement vocabularies than children in the control arm and reported greater participation in activities aside from circus. The fact that children improved motor competencies not directly related to circus per se, and that are standard for physical education delivery, suggested circus arts instruction may be particularly good at facilitating the transfer of skills, while offering students a fun, engaging and novel alternative to standard physical education approaches. Collectively, the results suggested that circus arts instruction in the context of physical education might be an effective way of supporting physical literacy development across a number of elements. It is possible that these approaches to physical literacy and learning could grow from approaches grounded in other disciplines that use a physical embodied approach to achieve outcomes.

To an outside observer, circus arts instruction in practice is free-flowing and group-based, with children participating at different stations all at the same time. Children self-select where to begin and how long they will stay at any one activity; they work independently on refinement of skills, often in small groups; the teacher moves freely between stations encouraging and providing support. ‘Moving together in this way’, coupled with the inherent performance nature of circus (‘playing to an audience’) may reduce social inhibition and encourage appropriate risk taking and experimentation. Learning to perform in this context for an audience rather than in front of the audience [37], both in training (learning) and in performance, is one core way in which an art-based approach differs from a sport-based approach. While it is true that sport is often played in front of others, the skills and structure of the games do not consider the audience as an inherent part of the process (it is at best a by-product). One possibility is that explicitly preparing for an audience may positively influence the development of physical literacy, and how other creative arts that are also equally concerned with ‘performance in front of others’ might also be useful in this context. We suggest that the field of actor training may bring important understanding in how to improve physical literacy in all age groups from this holistic perspective.

## 7. Actor Training

Physical practice is a core element in actor training. Habits and abilities are both developed and transformed through iteration and repetition with a distinct intention to expand the interior world of the performer [5]. This practice fosters simultaneous awareness of sensory stimulus, physical effort, technique and endurance and of imagination, language and feeling. An actor training approach assumes a nuanced understanding of the physical body in relation to the environment—an understanding in which senses, perception, imagination and physical actions are developed and remain archived in the body through a process of embodied cognition [42,43].

The term ‘actor training’ speaks to a breadth of practices that have become recognised over the twentieth century. Actor training processes are repeated and fundamentally physically embodied, which means they are usually developed with a group of people ‘on the floor’, working in a studio or rehearsal room, executing physical actions, vocal exercises, perceptual tasks and so on.

For the purposes of this paper, we are constraining the activities of actor training to the practices of Stanislavski [44]: methods of embodied imagination, the physical theatre approaches articulated by renowned clown/mime teacher Lecoq [45], the ‘Efforts’ of Laban’s Movement Analysis described by Bartenieff [46], and US theatre director Bogart’s [47] large ensemble improvisational training practices. The emphasis of these activities is on developing an extraordinary ‘extra-daily’ repertoire for the use of performance.

A prominent articulation of this idea occurs in Barba’s foundational work ‘The Paper Canoe’ [48], in which he explicates the notion of ‘extra daily activities’ as a pathway for action. He refers to this as ‘sats’: a visible state of readiness, preparation and aliveness in the body of the actor in the moment just before action cited in [47]. Actor training practices, although varied and stemming from different sources, share a number of characteristics. These practices are holistic and draw on kinesthetic, linguistic and imaginative processes. They are practiced regularly, over time, for incremental awareness, flexible mastery and technical proficiency (in the way that we might practice a musical instrument), and often begin with physical, non-verbal activities and introduce abstractions such as space and time, relationship and language over time. Actor training proposes activities as tasks, and builds layers of complexity. Such activities place the participants at the centre of the process, making space for their diverse cultures, abilities and capacities. Practices are embodied and physical (i.e., not sitting around a table reading scripts), and are often grounded in martial arts training, non-Western codified physical forms, and/or other somatic methodologies. These practices draw on understandings of embodiment, i.e., that the characteristics of the human world such as the surfaces we move on are associated with the capacities of our own bodies and the skill in moving we have acquired [49].

## 8. Mental Models of Actor Training

Recent interventions in the physical activity field have linked literacy with movement. For example, one experimental study with pre-schoolers had separate arms targeting movement skill instruction, free play and literacy (reading circle). Another study took this idea a step further by beginning with a theoretical premise that motor skill activities help with cognition, and then describing a movement and story-telling intervention in which the researchers observed student development by adding a storytelling activity to the physical activity class [50]. This fits with the notion of ‘thinking movement’ as described by Diamond [51], and discussed with reference to physical literacy in an early childhood context [52]. Alternatively, language, literacy and drama educators work from ‘story’ as a beginning point. These educators know how good story is for language development. They might approach interventions by adding movement training and a focus on motor skill development to see if it improves language development. Commonly, this might be an activity in which words are paired with action to assign meaning. Rieg and Paquette [53] referred to this as the ‘total physical response’ approach, and argued that it offers strategies that ‘enable classroom teachers to include kinaesthetic experiences’ in support of language learning as a physical process [53]. Yet there is little insight offered into how these activities operate over the longer term, or might be fundamental to social, cognitive and psychological development.

Both fields, in other words, approach motor and language development from a discipline-specific perspective. Also, it follows that both fields may learn from each other. This paper focuses on the pedagogy of actor training and the potential of its methodology to contribute to the discussion. The actor training methodologies we are showcasing adopt the notion of practice as a valuable contribution to somatic and kinaesthetic modalities which expand the internal space for and the valuing of embodied cognition. Actor training does not merely address the physical domain, nor is it just about story. Actor training encompasses a different cognitive capability, which could broadly be defined as embodied cognition. Such principles are also used in Gestalt therapy, in that it is considered important to understand how the structure of the body relates to emotions. From this perspective it is important to understand the body support from a biomechanical view and how this links not only to physical development, but also character [54]. The work of neurologist Damasio [55] indicated that pre-expressive cognition in children develops fundamentally: we are already forming stories before we can tell them. Stories can be organised visually, kinaesthetically, perceptively, or in other ways. An interdisciplinary philosopher [56] described this process as one in which perception leads meaning, and it is at this ‘molecular level’ where actor training can contribute. Over time, acting and performance practice finesses alternative mental models from which to interpret the world, such as: being aware of what you’re perceiving and how it makes your body respond; developing virtuosity in choosing responses; and expanding the embodied/spatial area inside you to have simultaneous attentions or to switch attentions—to have attention hierarchies. All this can draw into the larger embodied cognitive schema that an individual has constructed over a lifetime [42,56,57].

## 9. How Is Actor Training Relevant to Physical Literacy Programs

Blair [57] argued that the complexities in the demands on the training actor heighten their capacity to respond, create, initiate and to learn. The nature of actor training practices as task-based, iterative, and occurring over time affords a scaffolded, self-regulatory schema for the learner that intervenes on itself in a feedback loop of cognitive, social, affective and physical intervention for an individual [56,57]. Blair [57] reported on her experience of memorising a script, and the way she also developed a physical score (a set of prescribed movements or gestures) that she used when she performed that text. Such stories illustrate how actor training enables actors to develop and then harness strategies and non-verbal schemas that help them to package and organise narratives, memories, imaginative material, and other information.

Two of the authors of this paper have been applying these modalities within their own professional practices for many years. Our unique insights suggest that encounters with actor training offer additional specificity to motor/linguistic development approaches, and bring exciting new learning perspectives to the four domains of physical literacy identified by Sport Australia. An ongoing practice of actor training can deliver a number of benefits well beyond its remit, providing major health benefits, building connection, and developing empathy [7,58]. We have found that their own long-term actor training practice has enacted an aggregation of perceptual acuity, almost like a virtuosity of perception, that is, active fluency in nonverbal domains of affect, feeling, emotion and gesture whilst engaged with deep task absorption, which informs their micro-decision making toward optimising embodied responsiveness in daily life.

Theatre is a social practice and is frequently used in social interventions, in communities, in place-based interventions, with people with disability, and in therapeutic contexts. It also manifests in physical and cognitive practices such as education, aged care, and the rehabilitation of head injury, because of the affordances in actor training to develop cognitive skill that is not always linguistic. We might call this a ‘non-word space’ that is codified in some way by the individual—something that they know but are not quite sure how to talk about. The individual might imagine an idea, but not in images—it could be in feelings, or sensations, or a combination of those things. This ‘non-word space’ might manifest in a need to get up and demonstrate an idea physically.

Leading North American theatre director Anne Bogart has worked with trainee performers for decades, and conceptualises the value of the long-term iterative practice as an extension of what we might term empathy. She indicates that empathy necessitates ‘an investment of time, of single-minded concentration and of imagination’, and, like relationship-building, empathy requires ‘the ability to let go of our own world view long enough to be intrigued and moved by someone else’s’ [58]. Sofia’s study with actor training in the treatment of Parkinson’s Disease found that the motor control and ability required to complete some exercises coupled with the demand to manage the social situation resulted in a stabilising of the body schema of the individual while they focussed solely on their intention to move rather than an explicit motor act [7]. Performance studies scholar Lutterbie [59] investigated the way in which the individual’s physical gestures might lead in an expanded interior space for meaning-making in university acting students. Examples of such actor training activities included: working in silence with one another, walking, changing direction or velocity, shifting eye gaze and/or the action of looking toward and away, and incorporating, repeating and copying everyday gestures. These activities support individuals to demonstrate a greater range of tolerance for diverse meanings, to generate their own multiple meanings, and to appear more comfortable with not-knowing, or understanding the intention behind what a gesture might mean. Lutterbie claimed these findings offered evidence that acting practice ‘supports a holistic interplay between the external world and the internal one’ [59] and supported a deeper understanding of what we might identify as ‘the embodied human agent’.

## 10. Conclusions

The aim of this piece was to raise awareness of another player (pun intended) in the physical literacy literature considerations. Based on Australian interpretations of physical literacy being a construct of integrated physical, cognitive, social, and psychological learning [14], there are many synergies and opportunities for the advancement of the arts. Just as education, sport, and health are realizing the potential of physical literacy for inter-agency collaboration [19], the arts that express the artefacts of their work in the form of human movement would benefit from participating in this movement. In fact, thespian inspired movement patterns may provide us with additional insight to cognitive and psychological learning domains that remain entirely absent from the existing physical literacy and wider human movement literature.

Evidence we have reviewed herein suggests that the highly desirable transfer of learning between contexts and tasks may be facilitated by engaging in performing arts such as circus skills and actor training. We have outlined how circus- and actor-training strategies are compatible with the concept of physical literacy and could provide a way for physical literacy programs to incorporate learning in all four domains of physical literacy. The next step is to develop physical literacy programs with actor training content. This may require research methods to converge in creative new ways, as well as the concepts. For example, how could we design interventions that spanned sport/exercise activities and actor training? How could we look for and detect ‘far transfer’ of learning between contexts? Could methods developed in other contexts such as Gestalt therapy [54], help us to understand movement based learning in a physical literacy context? The scope of future collaborative work between disciplines is exciting and a fitting approach for a construct that is deemed holistic. Of course, this need not be limited solely to acting. Many of the essential elements of actor training that cover the core domains of physical literacy, including learning to play ‘for’ an audience, are generalizable to other contexts such as music performance, circus as we noted, and of course dance. All of these performance-based arts provide rich contexts for the exploration and development of physical literacy. As a fitting conclusion, we turn to Whitehead, who concluded her highly influential tome with a quote from a French phenomenological philosopher, Merleau-Ponty (1968, p. 429), that “our embodiment is indeed an art hidden in the depths of the human soul” [2]. As such, our journey towards physical literacy needs to recognise the true contribution of art.
